# Association between unrealistic comparative optimism and self‐management in individuals with type 2 diabetes: Results from a cross‐sectional, population‐based study

**DOI:** 10.1002/hsr2.157

**Published:** 2020-05-05

**Authors:** Florian M. Karl, Rolf Holle, Lars Schwettmann, Annette Peters, Christa Meisinger, Ina‐Maria Rückert‐Eheberg, Michael Laxy

**Affiliations:** ^1^ Institute of Health Economics and Healthcare Management Helmholtz Zentrum München (GmbH) Neuherberg Germany; ^2^ German Center for Diabetes Research (DZD) Neuherberg Germany; ^3^ Department of Medical Informatics, Biometry and Epidemiology University of Munich Munich Germany; ^4^ Institute of Epidemiology Helmholtz Zentrum München, GmbH, German Research Center for Environmental Health Neuherberg Germany

**Keywords:** accuracy, adherence, health behavior, health belief model, heart attack, myocardial infarction, optimistic bias, risk communication

## Abstract

**Background and aims:**

Unrealistic comparative optimism (UO), as the erroneous judgement of personal risks to be lower than the risks of others, could help explain differences in diabetes self‐management. The present study tested the hypothesis that individuals with type 2 diabetes who underestimate their comparative heart attack risk, have a lower adherence regarding recommended self‐management.

**Methods:**

We used data from individuals with type 2 diabetes participating in the German KORA (Cooperative Health Research in the Region of Augsburg) GEFU 4 (self‐administered health questionnaire 2016) study. UO was estimated by comparing participants' subjective comparative risk for having a heart attack within the next 5‐years (ie, “higher than others,” “average,” “lower than others”), with their objective comparative 10‐year cardiovascular disease risk based on the Framingham equations. We estimated binary logistic and linear regression models to analyze which characteristics were associated with UO and to test the association between UO and participants' self‐management behaviors (ie, regular self‐monitoring of body weight, blood sugar, and blood pressure, regular foot care, keeping a diabetes diary, and having a diet plan), and their sum score, respectively. All models were adjusted for socio‐demographic and disease‐related variables.

**Results:**

The studied sample included n = 633 individuals with type 2 diabetes (mean age 70.7 years, 45% women). Smokers and males were more likely to show UO than nonsmokers and females. Furthermore, a higher blood pressure and a higher body mass index were associated with a higher likelihood of UO regarding heart attack risk. However, UO was not significantly associated with patient self‐management.

**Conclusions:**

Unfavorable health behavior and risk factors are associated with UO. However, our results suggest that UO with regard to perceived heart attack risk may not be a relevant factor for patient self‐management in those with type 2 diabetes.

## 1 BACKGROUND

Type 2 diabetes is a major health concern worldwide and causes enormous societal costs.^1^, ^2^ Previous studies have shown that good self‐management can help slow down progression of the disease, prevent the occurrence of comorbidities,^3^, ^4^, ^5^ reduce mortality, and increase health‐related quality of life.^6^, ^7^

Unrealistic comparative optimism (UO) has been frequently suggested as a promising construct to explain health behavior and adherence in healthy and unhealthy individuals, and to ultimately tailor and improve interventions.^8^, ^9^ UO describes the tendency for people to make the erroneous assumption that they are less likely than others to experience a negative (health) event, for example, a heart attack.^9^, ^10^, ^11^ The personal risk perception, relative and absolute, has been identified as a relevant factor for explaining preventive behavior.^12^ Furthermore, other authors have reported that UO plays a role in all factors included in the Health Belief Model.^8^ Therefore, UO might help explain differences in preventive behaviors, for example, self‐management in patients with type 2 diabetes.^8^, ^9^ As Shepperd et al described, it is expected that individuals who show UO would show less preventive behaviors, that is, self‐management.^13^

In individuals with type 2 diabetes, the risk for a wide range of cardiovascular disease (CVD) is about 2‐fold compared to individuals without diabetes.^14^ Indeed, myocardial infarction (MI) accounts for more than 50% of all death in individuals with type 2 diabetes.^15^ Therefore, an accurate risk perception with regard to MI is especially important for individuals with type 2 diabetes. Studies analyzing UO regarding MI on an individual level are uncommon and mainly concentrate on predictors of UO.^13^ For example, Avis et al found that higher education was associated with a lower probability for UO.^16^ Furthermore, Radcliffe and Klein reported that disease‐specific education was associated with a lower probability for UO.^17^ Moreover, Ayanian and Cleary found that smokers older than 64 years were more likely to show UO regarding their MI risk than smokers younger than 64 years.^18^ In contrast, Strecher et al reported that young smokers (18‐29 years), individuals with lower education levels, and females were more likely to show UO, compared to smokers older than 29 years, individuals with higher education levels, and males, respectively.^19^

There have been few studies that have investigated the association between UO and health behavior where UO was determined by comparing a subjective and an objective risk, on an individual level.^13^ In a study that is unrelated to diabetes and heart attack risk, Dillard et al reported higher rates of unpleasant alcohol‐related events, for example, hangover or memory loss, among unrealistically optimistic individuals.^20^ We found no studies on the association between UO and self‐management in individuals with type 2 diabetes.

In this study, we measured individual‐level UO with regard to the risk of suffering a MI with a method that is very similar to the way it has been proposed by Avis et al.^16^ We compared participants' comparative risk judgments for having a heart attack (ie, “higher than that of other patients with type 2 diabetes of the same age,” “about the same as that of other patients with type 2 diabetes of the same age,” “lower than that of other patients with type 2 diabetes of the same age”) with their objectively calculated individual comparative risk of having a CVD based on the Framingham risk equations. Subsequently, we examined the characteristics associated with UO, and tested the hypothesis that individuals who show UO have a lower adherence rate with regard to recommended self‐management, in a sample of individuals with type 2 diabetes.

## 2 METHODS

### 2.1 Data source

**FIGURE 1:**
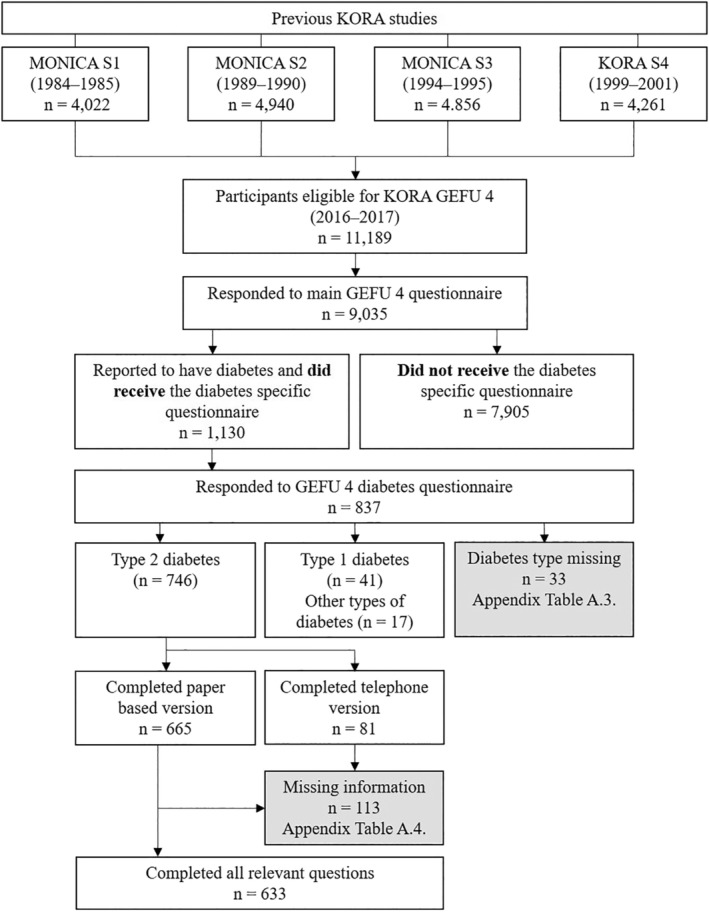
Participant flow diagram

We used data from the German KORA (Cooperative Health Research in the Region of Augsburg) GEFU 4 study (self‐administered health questionnaire 2016). KORA is a regional research platform that was established to conduct population‐based surveys and subsequent follow‐up studies in the fields of epidemiology, health economics, and health care research.^21^, ^22^ GEFU 4 was a cross‐sectional postal survey conducted from 2016 to 2017. The final analysis data set included n = 633 individuals with type 2 diabetes. A respective flow diagram is presented in Figure 1.

### 2.2 Overview on the assessment of UO in the literature

The general approach to measure UO starts with measuring comparative risk perception. The comparative risk perception is assessed by asking individuals to rate their personal risk of experiencing an event of interest relative to an appropriate peer. These ratings can be assessed with either direct or indirect methods.^9^, ^10^

For the direct approach, participants are asked whether they consider themselves more likely, equally likely, or less likely to experience a certain event in comparison with their peers, for example, age group.^10^ On a group level, the assumption is that if the mean comparative risk judgement of a group is below average, then this group shows UO at a group level.^9^ For example, Weinstein used the direct approach and identified a lack of experience regarding the outcome of interest as a main predictor of UO at a group level.^23^ However, this approach allows no conclusion about UO at an individual level.^9^

The indirect approach combines two items. First, the participants are asked to rate their personal likelihood of experiencing the event of interest, and second, to rate the likelihood of experiencing the event of interest for the average person within their peer group. The difference score between both responses is considered the amount of comparative optimism or pessimism, respectively.^10^ For example, Kim and Niederdeppe used an indirect approach and reported that comparative optimism had a moderating role in predicting intention to self‐protect against H1N1.^24^

Both the direct and the indirect approach, however, do not account for the actual individual‐level risk of people. Hence, they do not determine whether the individuals' comparative judgments are actually unrealistic.^10^ This can only be examined with the use of an objective comparator.^9^, ^10^ In other words, participants' estimates of whether they are equally likely, less likely, or more likely than others to experience a specific event, need to be compared with an objective comparator in order to test UO on an individual level. In health research, epidemiological risk equations are a practical option to measure people's objective risk to experience a specific event.^9^, ^10^, ^16^, ^17^, ^25^

### 2.3 Assessment of UO

We assessed UO using procedures modeled after the approach of Avis et al.^16^

First, we assessed the individuals' self‐rated risk in comparison with other patients of their age with type 2 diabetes with the following question: “Do you believe that your personal risk of suffering a heart attack within the next 5 years is higher than that of other patients with type 2 diabetes of your age?” The response categories were: (a) “yes, I believe my personal risk is higher,” (b) “I believe my risk is about the same,” and (c) “no, I believe my risk is lower”.

Second, in order to be able to compare the individuals' self‐rated comparative risk with their actual comparative risk, we calculated the “office‐based” Framingham risk (%), as described by D'agostino et al.^26^ The score uses age, sex, body mass index (BMI), systolic blood pressure distinguished by treatment status, smoking status, and diabetes status to estimate the individual 10‐year risk of suffering a CVD.^27^

**FIGURE 2:**
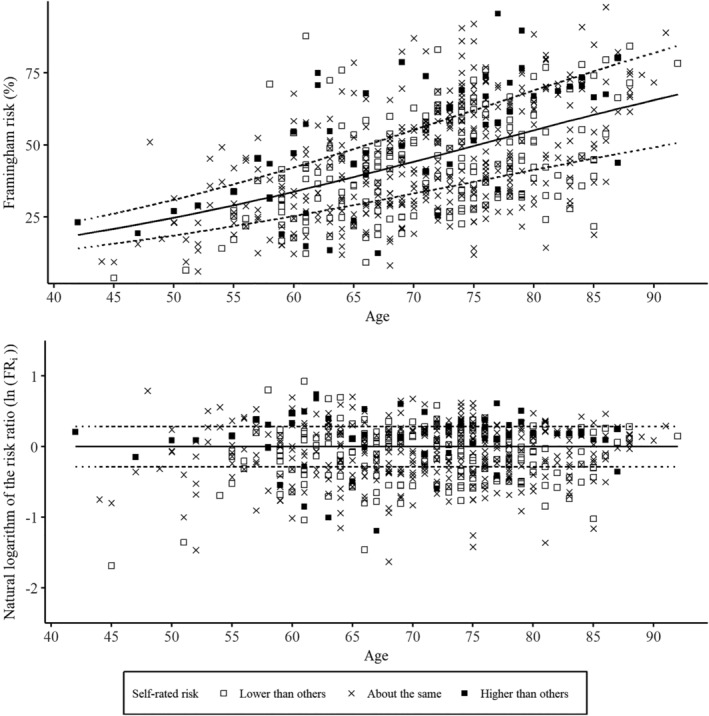
Distribution of calculated Framingham risks and cut‐offs for UO and unrealistic comparative pessimism (UP). The upper part of the Figure shows the calculated Framingham risk (*F*_i_) plotted for every individual. The solid line represents the mean risk prediction dependent on age (*FP*_i_). The dotted lines show the nonlogarithmic cut‐offs for the risk ratio (*FR*_i_) between *F*_i_ and *FP*_i_. The lower part of the Figure shows the natural logarithm of the risk ratio (ln(*FR*_i_)) for every individual. The solid line represents no difference (ln(1)) and the dotted lines represent the cut‐offs for ln(*FR*_i_), that is, below average (ln(0.75)) and above average (ln(1.33))

Third, we calculated the ratio (*FR*_i_) of each individual's calculated Framingham risk (*F*_i_) and the mean calculated risk of people of the respective age (*FP*_i_). The *FP*_i_ was estimated using a pseudo‐binomial approach, calculating a binomial regression with logit link based on the distribution of calculated Framingham risks in our sample (*FP*_i_ *=* exp(*β*_0_ *+* age_i_ × *β*_age_). *FP*_i_ was only regressed on age because participants were instructed to state their risk relative to other people of their *age* with diabetes. As described by Avis et al., we used the natural log transformation of the calculated ratio (ln(*FR*_i_)) and the cut‐offs ln(0.75) and ln(1.33) in order to create a symmetric distribution and equal “risk distances”.^16^ See Figure 2.

Individuals with ln(*FR*_i_) < ln(0.75) were considered to have a risk below average, and individuals with ln(*FR*_i_) *>* ln(1.33) were considered to have a risk above average. Finally, we compared the self‐rated risk with the calculated risk category.^16^ When individuals self‐rated their comparative risk as below average but their calculated comparative risk was average or above average, they were grouped with UO. Moreover, when individuals self‐rated their comparative risk as average but their calculated comparative risk was above average, they were also grouped with UO. For Unrealistic comparative pessimism (UP), the grouping was done accordingly. See Table 1 for an overview. Based on this approach, individuals with a low calculated risk (ln(*FR*_i_) *<* ln(0.75)) could not be grouped with UO, and individuals with a high calculated risk (ln(*FR*_i_) *>* ln(1.33)) could not be grouped with UP. To approach this conceptual limitation, we excluded individuals with a low calculated risk (ln(*FR*_i_) *<* ln(0.75)) and individuals with a high calculated risk (ln(*FR*_i_) > ln(1.33)) from all further analyses on UO (underestimation of comparative risk) and UP (overestimation of comparative risk), respectively. See Table 1 for an overview.

**TABLE 1:** Comparison between self‐rated and calculated comparative risk

Self‐rated risk	Objective relative risk		
	Below average	Average	Above average
“Lower than others”	n = 64 (Accurate)	n = 113 (UO)	n = 23 (UO)
“Average”	n = 110 (UP)	n = 203 (Accurate)	n = 66 (UO)
“Higher than others”	n = 9 (UP)	n = 29 (UP)	n = 16 (Accurate)

*Note*: The cells with colored background were excluded from some parts of the analysis. Specifically, individuals with an objective relative risk below average (lighter gray) were excluded from analyses regarding UO because per definition they could not be grouped with UO. Likewise, individuals with an objective relative risk above average (darker gray), were excluded from analyses regarding UP because per definition they could not be grouped with UP.

Abbreviations: UO, unrealistic comparative optimism; UP, unrealistic comparative pessimism.

### 2.4 Assessment of self‐management

Our measures of diabetes self‐management behavior were based on a compliance score introduced by Arnold‐Wörner et al.^28^ Within our study, we assessed the following self‐management behaviors: monitoring of body weight (at least once per week), conducting regular foot care (checking for wounds at least once per week), measuring blood sugar (at least once a day for patients treated with insulin and at least once a week for all others), measuring blood pressure (at least once per week), keeping a diabetes diary, and having a diet plan. We asked participants to consider the last 6 months for their answers ((a) “daily,” (b) “at least once per week,” (c) “once or twice per month,” (d) “less than once per month”). The respective cut‐off points were based on recommendations by the European NIDDM (noninsulin‐dependent diabetes mellitus) Policy Group^29^ and the American Diabetes Association.^30^ Furthermore, we combined the six self‐managing behaviors into a self‐management score. In this score, one point was attributed per criterion in every individual, as proposed by Arnold‐Wörner et al.^28^ A similar score has been shown to be highly predictive for all‐cause mortality in patients with type 2 diabetes.^7^

### 2.5 Covariates

To calculate the Framingham risk (%), we derived BMI from body height measured at the respective baseline study and self‐reported body weight at the time of GEFU 4. Age, sex, systolic blood pressure, blood pressure treatment status (medication), and smoking status were also based on self‐report at GEFU 4. Other than that, we assessed whether participants' treatment regimen included the injection of insulin, as we assumed treatment with insulin as an indicator for disease severity. Furthermore, we assessed education (primary education, ≤10 years of school; secondary/tertiary education, >10 years of school) and whether participants had ever participated in a diabetes education program that was not part of routine care or during a hospital stay. Finally, we asked participants whether they had ever had a heart attack that was diagnosed by a physician.

### 2.6 Statistical analysis

In a first step, we calculated frequencies and means with regard to measured characteristics and self‐management behaviors—overall and stratified by the three categories of self‐rated comparative risk, that is, “higher than others,” “average,” “lower than others”.

Second, we regressed the self‐rated comparative risk on the Framingham variables (ie, age, sex, systolic blood pressure, blood pressure treatment status, BMI, and smoking status) and the variables education, participation in a diabetes education program, treatment with insulin, and history of MI. Likewise, UO and UP were regressed on the same set of variables in two separate binary logistic regression models.

Finally, we estimated binary logistic regression models and ordinary least square regression models to test the association between individual‐level UO, UP, and the six measured self‐management behaviors and their sum‐score, respectively. We adjusted all models on the association with self‐management for age, sex, BMI, blood pressure treatment status, systolic blood pressure, smoking status, education, participation in a diabetes education program, treatment with insulin, and history of MI. Additionally, we adjusted all models for self‐rated risk. Thereby, we tried to disentangle the association between UO and self‐management behavior from confounding by positive or negative self‐view, that is, self‐rated risk “lower than others” or “higher than others”. As described by Humberg et al, the mere positivity of self‐view needs to be differentiated from the erroneous positive self‐view, that is, UO.^31^ A *P*‐value <.05 was considered to be statistically significant. Missing information in the items of the Framingham risk score was imputed using a predictive mean matching approach (see Table 2 for details).^32^, ^33^ Analysis was performed with R Studio^34^ and R 3.4.1 for Windows.

**TABLE 2:** Characteristics for the complete sample and self‐rated risk groups

	Total (n = 633)		Self‐rated risk					
			Lower than others (n = 200)		Average (n = 379)		Higher than others (n = 54)	
Framingham variables	n or mean	% or SD	n or mean	% or SD	n or mean	% or SD	n or mean	% or SD
Age	70.7	9.1	71.1	8.6	70.8	9.2	69.2	10.8
Male	349	55.1	112	56.0	199	52.5	38	70.4
Smoking (yes)	67	10.6	17	8.5	42	11.1	8	14.8
BMI (kg/cm^2^)	29.8	5.0	30.2	4.90	30.1	4.93	29.1	5.15
Blood pressure treatment (yes)	502	79.3	141	70.5	313	82.6	48	88.9
Systolic blood pressure (mmHg)	132.4	15.8	132.1	16.0	132.2	15.3	137.8	20.5
Framingham risk (%)	45.3	18.5	43.7	17.1	45.3	18.8	51.5	20.1
Covariates								
>10 years of schooling	260	41.1	94	47.2	150	39.6	16	29.6
Insulin therapy (yes)	127	20.1	36	18.2	76	20.1	15	27.8
D. education (yes)	336	53.7	94	47.2	206	55.2	36	66.7
MI history (yes)	66	10.4	18	9.00	32	8.4	16	29.6
Self‐management								
Self‐monitoring of body weight (≥1 per week = 1)	352	55.9	123	61.8	197	52.3	32	59.3
Wound checking (≥1 per week = 1)	348	55.9	116	58.9	200	53.8	32	59.3
Self‐monitoring of blood sugar (≥1 per week = 1 or ≥ daily when treated with insulin = 1)	235	40.8	76	41.3	140	41.1	19	37.3
Self‐monitoring of blood pressure (≥1 per week = 1)	305	48.8	100	50.8	180	48.1	25	46.3
Keeping a diabetes diary (yes = 1)	171	27.6	50	25.4	107	28.8	14	26.9
Having a diet plan (yes = 1)	57	9.2	20	10.2	30	8.2	7	13.2
Self‐management score (0‐6)	2.3	1.6	2.4	1.6	2.3	1.5	2.4	1.6

*Note*: The variables used for calculating the Framingham risk were essential to our study. Within the 633 individuals who self‐rated their comparative MI risk, we found 67 missing values for systolic blood pressure, 3 missing values for smoking status, and 11 missing values for BMI. In order to avoid loss of power for our analysis, we decided to apply a predictive mean matching approach, as introduced by Little^32^ within the variables that were relevant to the calculation of the Framingham risk. The imputation was performed with the R package “Mice”.^33^ The self‐management score was composed by adding the six self‐managing behaviors into a single score, in which one point was attributed per criterion in every individual (See Methods).

Abbreviations: D. education, diabetes education program (yes); MI, myocardial infarction.

### 2.7 Sensitivity analysis

The Framingham risk is supposed to be calculated only for individuals <75 years of age and without a prior CVD. Therefore, we excluded individuals >74 years or with a history of MI in our first sensitivity analysis (n = 356).

In our second sensitivity analysis, we approached the issue that individuals might have compared themselves within their sex, even though the question did not imply this. Therefore, we estimated the mean risk (*FP*_i_) in a binomial regression based on age and sex (*FP*_i_ *=* exp[*β*_0_ *+* age_i_ *× β*_age_ + sex_i_ *× β*_sex_]). We then tested the association between UO and the assessed characteristics, as well as the association between UO and self‐management similar to our main analysis.

In further sensitivity analyses, we examined the association between UO and self‐management using different cut‐offs for the calculated risk ratio ln(*FR*_i_). We tested very sensitive cut‐offs, that is, ln(0.86) < ln(*FR*_i_) > ln(1.16), and very specific cut‐offs, that is, ln(0.60) < ln(*FR*_i_) > ln(1.66)).

Finally, multiple previous studies did not exclude individuals with a low comparative risk or a high comparative risk from analysis including UO or UP, respectively. Therefore, in another sensitivity analysis, we repeated our main analysis without the exclusion of individuals based on their objective comparative risk.

### 2.8 Ethical considerations

The study was approved by the Bavarian Medical Association (approval number: 08064). All procedures performed in studies involving human participants were in accordance with the ethical standards of the ethics committee of the Bavarian Medical Association and with the 1964 Helsinki Declaration and its later amendments or comparable ethical standards. Informed consent was obtained from all individual participants included in the study.

## 3 RESULTS

### 3.1 Study sample

Of 9035 individuals who participated in the KORA GEFU 4 study, 1130 individuals reported to have any type of diabetes. The final analyzed sample included information from 633 individuals with type 2 diabetes, with a mean age of 70.7 years (SD = 9.1 years), 55% of which were males. Details are shown in Figure 1 (see also Appendix Table A3). The mean self‐management score was about the same in all groups of self‐rated risk. All details on the analyzed characteristics are shown in Table 2.

Characteristics of individuals with missing information regarding their self‐management or their self‐rated heart attack risk (n = 113) are reported in Appendix Table A4. Individuals with missing information were more likely to smoke and less likely to have higher education compared to individuals without missing information.

### 3.2 Associations between the individuals' characteristics and self‐rated risk, UO, and UP

Overall, 32% of the participants (200 of 633) rated their MI risk lower than that of others, while only 9% (54 of 633) rated their risk higher than that of others. Males and individuals with a history of MI were more likely to self‐rate themselves with a higher than average risk of suffering a MI in the future than females and individuals without a history of MI, respectively (Table 3). Individuals treated for high blood pressure were less likely than individuals without blood pressure treatment to self‐rate their risk lower than that of other type 2 diabetes patients of their age (Table 3).

**TABLE 3:** The association between UO, UP, and the individuals' characteristics in the main analysis

	(1) “Lower than others” (n = 200)		(2) “Higher than others” (n = 54)	
	Unadjusted odds ratio[95% CI]	*P*‐value	Unadjusted odds ratio[95% CI]	*P*‐value
Age (divided by10)	1.06 [0.86; 1.31]	.585	0.80 [0.56; 1.15]	.225
Male sex	0.92 [0.64; 1.31]	.631	2.07 [1.11; 4.02]	.025
Smoking (yes)	0.70 [0.37; 1.26]	.246	1.47 [0.58; 3.36]	.388
BMI	0.98 [0.94; 1.01]	.203	1.01 [0.95; 1.07]	.794
Blood pressure treatment	0.49 [0.32; 0.74]	.001	1.67 [0.71; 4.63]	.272
Blood pressure	1.00 [0.98; 1.01]	.463	1.01 [1.00; 1.03]	.064
>10 years of schooling	1.36 [0.95; 1.95]	.092	0.55 [0.28; 1.02]	.064
Insulin therapy (yes)	1.01 [0.63; 1.60]	.969	1.24 [0.60; 2.45]	.545
Diabetes education program (yes)	0.74 [0.51; 1.06]	.103	1.41 [0.75; 2.71]	.288
MI history	0.93 [0.50; 1.67]	.813	3.89 [1.91; 7.73]	<.001

	(3) UO (n = 202)		(4) UP (n = 148)	
	Unadjusted odds ratio [95% CI]	*P*‐value	Unadjusted odds ratio [95% CI]	*P*‐value
Age (divided by 10)	1.20 [0.93; 1.55]	.163	0.57 [0.43; 0.75]	<.001
Male sex	4.84 [2.78; 8.68]	<.001	0.11 [0.06; 0.19]	<.001
Smoking status	4.82 [2.46; 9.79]	<.001	0.25 [0.09; 0.62]	.004
BMI	1.05 [1.00; 1.10]	.037	0.97 [0.92; 1.01]	.157
Blood pressure treatment	1.26 [0.70; 2.27]	.439	0.72 [0.43; 1.23]	.231
Blood pressure	1.04 [1.02; 1.05]	<.001	0.95 [0.93; 0.96]	<.001
>10 years of schooling	1.26 [0.83; 1.91]	.278	0.74 [0.46; 1.17]	.202
Insulin therapy (yes)	0.92 [0.54; 1.56]	.765	0.91 [0.49; 1.67]	.769
Diabetes education program (yes)	0.89 [0.58; 1.35]	.582	1.17 [0.74; 1.87]	.498
MI history	0.52 [0.27; 0.98]	.049	2.17 [1.03; 4.52]	.039

*Note*: The association of patient characteristics with low comparative risk perception, high comparative risk perception, UO, and UP was examined in four binary logistic regressions (1 through 4). In (1), participants with average and high comparative risk perception were used as reference to the participants with a low comparative risk perception. In (2), participants with average and low comparative risk perception were used as reference to the participants with a high comparative risk perception. In (3), participants at average or high objective comparative risk and who were not grouped with UO were used as reference to participants with an average or high objective comparative risk but who were grouped with UO. In (4), participants at low or average objective comparative risk and who were not grouped with UP were used as reference to participants with a low or average objective comparative risk but who were grouped with UP.

Abbreviations: UO, unrealistic comparative optimism; UP, unrealistic comparative pessimism.

Within the studied sample, 32% of individuals (202 of 633) showed UO—that is, have a higher or equal calculated Framingham risk compared to other patients with type 2 diabetes of the same age but think their risk is average or lower than average, respectively. On the other hand, 23% (148 of 633) showed UP—that is, have a lower or equal calculated Framingham risk compared to other patients with type 2 diabetes of the same age but think their risk is average or higher than average, respectively (Table 1). Males, smokers, individuals with a higher BMI, a higher blood pressure, and no history of MI were more likely than females, nonsmokers, individuals with a lower BMI, lower blood pressure, and no history of MI, to underestimate their comparative risk, that is, to show UO (Table 3). Accordingly, males, smokers, individuals with a higher blood pressure, and individuals with no history of MI were less likely than females, nonsmokers, individuals with a lower blood pressure, and individuals with a history of MI, to overestimate their comparative risk, that is, to show UP (Table 3). Furthermore, older individuals were less likely than younger individuals to show UP.

### 3.3 Association between UO, UP, and the participants' self‐management

Overall, we found no statistically significant association between the measured UO or UP and the six self‐management behaviors (see Tables 4 and 5). However, the association of UO with self‐management (Table 4, model 2) was predominantly negative in its direction (OR < 1), while the association of a positive self‐view, that is, rating the personal risk lower than that of others, with self‐management was predominantly positive (OR > 1).

**TABLE 4:** Association between UO and the participants' self‐management

(n = 450)	Regular self‐monitoring of body weight^a^		Wound checking^a^		Regular self‐monitoring of blood sugar^a^	
	OR [95% CI]	*P*‐value	OR [95% CI]	*P*‐value	OR [95% CI]	*P*‐value
Model 1						
UO	0.64 [0.37; 1.12]	.121	1.06 [0.61; 1.87]	.827	1.13 [0.62; 2.04]	.682
Self‐view						
Average	0.98 [0.51; 1.87]	.941	0.75 [0.39; 1.44]	.394	1.14 [0.58; 2.30]	.707
Positive	1.70 [0.70; 4.11]	.236	0.94 [0.39; 2.28]	.898	1.20 [0.48; 3.07]	.697
Model 2						
UO	0.66 [0.32; 1.35]	.26	0.68 [0.32; 1.47]	.334	0.71 [0.29; 1.71]	.451
Self‐view						
Average	1.07 [0.52; 2.16]	.861	1.04 [0.50; 2.18]	.911	2.03 [0.87; 4.97]	.11
Positive	1.78 [0.61; 5.24]	.292	1.98 [0.63; 6.32]	.243	4.18 [1.11; 16.48]	.037

	Regular self‐monitoring of blood pressure^a^		Keeping a diabetes diary^a^		Having a diet plan^a^		Sum‐score^b^	
	OR [95% CI]	*P*‐value	OR [95% CI]	*P*‐value	OR [95% CI]	*P*‐value	β [95% CI]	*P*‐value
Model 1								
UO	0.58 [0.33; 1.03]	.064	1.20 [0.63; 2.21]	.568	0.80 [0.22; 2.32]	.708	−0.26 [−0.72; 0.20]	.273
Self‐view								
Average	1.12 [0.59; 2.15]	.729	1.00 [0.48; 2.21]	.995	0.64 [0.23; 2.05]	.407	0.00 [−0.53; 0.53]	.999
Positive	2.15 [0.89; 5.23]	.091	0.80 [0.30; 2.24]	.665	1.13 [0.25; 6.10]	.878	0.37 [−0.34; 1.09]	.308
Model 2								
UO	0.57 [0.27; 1.18]	.133	0.73 [0.29; 1.76]	.481	0.35 [0.08; 1.30]	.136	−0.45 [−1.00; 0.10]	.107
Self‐view								
Average	1.32 [0.64; 2.73]	.457	1.63 [0.66; 4.29]	.303	0.99 [0.32; 3.60]	.992	0.26 [−0.26; 0.79]	.324
Positive	2.86 [0.95; 8.73]	.063	2.25 [0.58; 9.26]	.249	3.71 [0.60; 27.42]	.176	0.94 [0.12; 1.76]	.025

*Note*: Model 1 included the variables UO and self‐view; Model 2 included UO, self‐view, age, sex, BMI, blood pressure treatment status, systolic blood pressure, smoking status, education, participation in a diabetes education program, treatment with insulin, and history of MI. In the analysis for Table 4, we only included individuals with an average or comparatively high Framingham risk (n = 450).

Abbreviation: UO, unrealistic comparative optimism.

a Binary logistic regression analysis.

b Linear regression analysis.

**TABLE 5:** Association between UP and the participants' self‐management

(n = 528)	Regular self‐monitoring of body weight^a^		Wound checking^a^		Regular self‐monitoring of blood sugar^a^	
	OR [95% CI]	*P*‐value	OR [95% CI]	*P*‐value	OR [95% CI]	*P*‐value
Model 1						
UO	0.90 [0.56; 1.43]	.654	1.48 [0.92; 2.39]	.108	1.03 [0.62; 1.69]	.913
Self‐view						
Average	0.73 [0.48; 1.10]	.138	0.77 [0.51; 1.15]	.197	0.99 [0.65; 1.53]	.976
Positive	1.14 [0.49; 2.75]	.759	0.69 [0.29; 1.64]	.396	0.81 [0.32; 1.97]	.642
Model 2						
UO	0.83 [0.45; 1.53]	.554	1.59 [0.86; 2.98]	.142	1.45 [0.72; 2.93]	.304
Self‐view						
Average	0.73 [0.47; 1.14]	.171	0.70 [0.45; 1.10]	.127	0.76 [0.45; 1.27]	.296
Positive	1.05 [0.38; 2.96]	.920	0.54 [0.19; 1.51]	.239	0.32 [0.09; 1.02]	.058

	Regular self‐monitoring of blood pressure^a^		Keeping a diabetes diary^a^		Having a diet plan^a^		Sum‐score^b^	
	OR [95% CI]	*P*‐value	OR [95% CI]	*P*‐value	OR [95% CI]	*P*‐value	β [95% CI]	*P*‐value
Model 1								
UO	0.83 [0.52; 1.33]	.449	1.53 [0.92; 2.55]	.100	1.45 [0.63; 3.27]	.372	0.11 [−0.29; 0.51]	.583
Self‐view								
Average	1.07 [0.71; 1.61]	.737	1.03 [0.64; 1.64]	.914	0.87 [0.41; 1.85]	.714	−0.12 [−0.46; 0.22]	.493
Positive	0.97 [0.41; 2.26]	.944	0.55 [0.20; 1.46]	.246	1.15 [0.28; 4.33]	.841	−0.23 [−0.95; 0.48]	.522
Model 2								
UO	0.97 [0.53; 1.79]	.923	1.84 [0.87; 3.89]	.11	1.46 [0.50; 4.26]	.485	0.27 [−0.19; 0.72]	.253
Self‐view								
Average	0.93 [0.59; 1.45]	.746	0.92 [0.52; 1.62]	.763	0.84 [0.37; 1.94]	.688	−0.27 [−0.60; 0.07]	.120
Positive	0.67 [0.24; 1.86]	.450	0.31 [0.08; 1.14]	.085	1.09 [0.20; 5.58]	.914	−0.67 [−1.43; 0.09]	.084

*Note*: Model 1 included the variables UO and self‐view. Model 2 included UO, self‐view, age, sex, BMI, blood pressure treatment status, systolic blood pressure, smoking status, education, participation in a diabetes education program, treatment with insulin, and history of MI. In the analysis for Table 5, we only included individuals with an average or comparatively high Framingham risk (n = 528).

Abbreviation: UP, unrealistic comparative pessimism.

a Binary logistic regression analysis.

b Linear regression analysis.

### 3.4 Sensitivity analysis

In the subset of individuals under 75 years of age and without a prior CVD, we found very similar associations as reported for our main analysis. (Appendix Table A1 upper half).

**APPENDIX TABLE A1:** The association between UO, UP, and the individuals' characteristics in the conducted sensitivity analyses

	Subsample of individuals with no MI history and <75 years of age			
	UO (n = 123)		UP (n = 85)	
	Odds ratio [95% CI]	*P*‐value	Odds ratio [95% CI]	*P*‐value
Age (divided by 10)	1.20 [0.93; 1.55]	.163	1.20 [0.93; 1.55]	.163
Male sex	4.84 [2.78; 8.68]	<.001	4.84 [2.78; 8.68]	<.001
Smoking (yes)	4.82 [2.46; 9.79]	<.001	4.82 [2.46; 9.79]	<.001
BMI	1.05 [1.00; 1.10]	.037	1.05 [1.00; 1.10]	.037
Blood pressure treatment	1.26 [0.70; 2.27]	.439	1.26 [0.70; 2.27]	.439
Blood pressure	1.04 [1.02; 1.05]	<.001	1.04 [1.02; 1.05]	<.001
>10 years of schooling	1.26 [0.83; 1.91]	.278	1.26 [0.83; 1.91]	.278
Insulin therapy (yes)	0.92 [0.54; 1.56]	.765	0.92 [0.54; 1.56]	.765
Diabetes education program (yes)	0.89 [0.58; 1.35]	.582	0.89 [0.58; 1.35]	.582

	Estimated mean risk calculated based on age and sex			
	UO (n = 200)		(4) UP (n = 102)	
	Odds ratio [95% CI]	*P*‐value	Odds ratio [95% CI]	*P*‐value
Age (divided by 10)	1.11 [0.88; 1.40]	.386	0.59 [0.44; 0.79]	<.001
Male sex	0.71 [0.49; 1.04]	.077	1.31 [0.80; 2.18]	.285
Smoking status	4.31 [2.38; 8.02]	<.001	0.25 [0.06; 0.82]	.034
BMI	1.01 [0.97; 1.05]	.658	0.95 [0.89; 1.00]	.068
Blood pressure treatment	0.85 [0.49; 1.48]	.562	0.35 [0.20; 0.60]	<.001
Blood pressure	1.01 [1.00; 1.02]	.097	0.93 [0.91; 0.95]	<.001
>10 years of schooling	1.40 [0.95; 2.06]	.087	0.51 [0.31; 0.85]	.011
Insulin therapy (yes)	1.12 [0.69; 1.81]	.633	1.03 [0.53; 1.92]	.934
Diabetes education program (yes)	0.74 [0.51; 1.09]	.131	1.32 [0.80; 2.21]	.283
MI history	0.79 [0.42; 1.44]	.457	2.84 [1.40; 5.62]	.003

*Note*: In model (1) and (3), participants at average or high objective comparative risk and who were not grouped with UO were used as reference to participants with an average or high objective comparative risk but who were grouped with UO. In model (2) and (4), participants at low or average objective comparative risk and who were not grouped with UP were used as reference to participants with a low or average objective comparative risk but who were grouped with UP.

Abbreviations: UO, unrealistic comparative optimism; UP, unrealistic comparative pessimism.

When the objective comparator was based on a comparison between the calculated individual risk and the mean risk of individuals of the respective age and sex, smoking and a higher blood pressure were still significantly associated with UO and UP. However, the associations between sex, BMI, and UO and UP were not statistically significant anymore. Detailed results are provided in the lower half of Appendix Table A1.

The results of the sensitivity analyses, like those in the main analysis, showed no consistent and statistically significant associations between UO and patient self‐management (Appendix Table A2).

**APPENDIX TABLE A2:** The association between UO, UP, and the individuals' self‐management in the conducted sensitivity analysis

	Regular self‐monitoring of body weight^a^	Wound checking^a^	Regular self‐monitoring of blood sugar^a^	Regular self‐monitoring of blood pressure^a^	Keeping a diabetes diary^a^	Having a diet plan^a^	Sum‐score^b^
	OR [95% CI]	OR [95% CI]	OR [95% CI]	OR [95% CI]	OR [95% CI]	OR [95% CI]	β [95% CI]
(1) No MI history & < 75 years of age							
UO	0.99 [0.38; 2.57]	2.07 [0.77; 5.60]	0.85 [0.24; 2.89]	0.82 [0.31; 2.14]	1.12 [0.32; 3.75]	0.38 [0.04; 2.54]	0.28 [−0.43; 0.99]
UP	1.22 [0.55; 2.74]	1.19 [0.53; 2.67]	1.33 [0.52; 3.43]	1.84 [0.83; 4.16]	1.97 [0.70; 5.65]	2.26 [0.52; 10.65]	0.50 [−0.10; 1.09]
(2) Estimated mean risk calculated based on age and sex							
UO	0.97 [0.47; 2.02]	0.58 [0.27; 1.24]	1.21 [0.51; 2.86]	0.98 [0.46; 2.06]	0.92 [0.35; 2.40]	0.74 [0.20; 2.64]	−0.09 [−0.63; 0.45]
UP	0.47 [0.23; 0.96]	1.18 [0.57; 2.42]	0.52 [0.22; 1.19]	1.08 [0.53; 2.23]	0.40 [0.15; 1.00]	2.68 [0.84; 8.39]	−0.39 [−0.94; 0.16]
(3) Specific cut‐offs							
UO	0.86 [0.26; 2.73]	1.25 [0.38; 4.34]	0.66 [0.16; 2.67]	1.30 [0.40; 4.25]	0.93 [0.22; 3.63]	0.90 [0.11; 4.93]	−0.10 [−0.97; 0.78]
UP	0.71 [0.34; 1.45]	1.55 [0.74; 3.31]	1.47 [0.62; 3.44]	1.07 [0.51; 2.23]	0.88 [0.35; 2.11]	5.73 [1.86; 17.50]	0.12 [−0.43; 0.67]
(4) Sensitive cut‐offs							
UO	1.01 [0.51; 1.99]	1.25 [0.60; 2.58]	1.36 [0.58; 3.23]	0.98 [0.49; 1.96]	0.74 [0.31; 1.75]	0.49 [0.13; 1.77]	0.34 [−0.19; 0.87]
UP	0.87 [0.44; 1.72]	2.03 [1.03; 4.04]	2.04 [0.91; 4.63]	1.28 [0.64; 2.53]	0.92 [0.39; 2.16]	0.67 [0.20; 2.28]	0.37 [−0.14; 0.87]
(5) No exclusion of individuals based on their calculated relative risk category							
UO	0.61 [0.35; 1.07]	0.91 [0.51; 1.60]	1.07 [0.56; 2.04]	0.75 [0.43; 1.32]	0.96 [0.48; 1.88]	0.81 [0.31; 2.05]	−0.20 [−0.61; 0.22]
UP	0.72 [0.48; 1.07]	1.19 [0.69; 2.06]	1.44 [0.76; 2.75]	1.01 [0.59; 1.75]	1.52 [0.79; 2.95	2.15 [0.85; 5.43]	0.07 [−0.34; 0.47]
(6) No exclusion of individuals based on their calculated relative risk category							
UO	0.67 [0.45; 1.01]	0.82 [0.55; 1.22]	1.25 [0.83; 1.90]	0.86 [0.57; 1.27]	0.96 [0.61; 1.50]	0.88 [0.43; 1.76]	−0.22 [−0.55; 0.10]
UP	0.98 [0.65; 1.48]	1.23 [0.81; 1.87]	0.94 [0.60; 1.45]	0.98 [0.64; 1.48]	1.23 [0.78; 1.94]	1.42 [0.68; 2.88]	0.06 [−0.29; 0.40]

*Note*: Sensitivity analysis (1) to (5) were adjusted for self‐view, age, sex, BMI, blood pressure treatment status, systolic blood pressure, smoking status, education, participation in a diabetes education program, treatment with insulin, and history of MI. Sensitivity analysis (6) was only adjusted for self‐view. In sensitivity analyses (1) to (4), where UO was the predictor of interest, we included individuals with an average or comparatively high Framingham risk. In sensitivity analyses (1) to (4), where UP was the predictor of interest, we included individuals with an average or comparatively low Framingham risk. Sensitivity analysis (5) and (6) included all participants.

Abbreviations: UO, unrealistic comparative optimism; UP, unrealistic comparative pessimism.

a Binary logistic regression analysis.

b Linear regression analysis.

**APPENDIX TABLE A3:** Characteristics of individuals with missing information on diabetes type

	Studied sample (n = 633)		Individuals with missing diabetes type (n = 33)	
	n or mean	% or SD	n or mean	% or SD
Age	70.7	9.1	70.8	10.6
Male	349	55.1	21	63.6
Smoking (yes)	67	10.6	4	12
BMI (kg/cm^2^)	29.8	5.0	28.9	4.3
>10 years of schooling	260	41.1	12	36.4

**APPENDIX TABLE A4:** Characteristics of individuals with missing information

	Studied sample (n = 633)		Individuals with missing data (n = 113)	
	n or mean	% or SD	n or mean	% or SD
Age	70.7	9.1	71.8	10.1
Male	349	55.1	57	50.4
Smoking (yes)	67	10.6	18	15.9
BMI (kg/cm^2^)	29.8	5.0	30.0	5.7
>10 years of schooling	260	41.1	36	31.9

## 4 DISCUSSION

In this study, we measured individual UO with regard to the risk of suffering a MI by comparing participants' comparative risk judgments for having a MI with the ratio between their calculated CVD risk and the mean CVD risk of people of their age. Subsequently, we examined the characteristics associated with UO, and tested the hypothesis that individuals who show UO have a lower adherence rate with regard to recommended self‐management in a sample of 633 individuals with type 2 diabetes.

We found that 32% of the participants in our study rated their personal MI risk lower than average compared with other individuals of their age with type 2 diabetes, while only 9% rated it higher. Moreover, individuals were about 1.4 times more likely to show UO than to show UP concerning their MI risk. Specifically, individuals with no history of MI, males, smokers, and individuals with a higher blood pressure were more likely than individuals with a history of MI, females, nonsmokers, and individuals with a lower blood pressure, to show UO. The associations of these characteristics with UP were reversed. Finally, in our main analysis, we did not observe a statistically significant association between UO and self‐management behavior.

The relatively high frequency of unrealistically optimistic responses compared to unrealistically pessimistic responses on a group level, as well as on an individual level, was not surprising. Similar results have been reported in previous studies,^16^, ^17^ and with respect to other negative events on a group level,^8^, ^11^, ^35^ and on an individual level.^25^, ^36^ One reason for the predominantly optimistic responses may be the person‐positivity bias.^9^, ^37^ Person‐positivity bias states that individuals dehumanize the “average person,” which leads to a worse rating of the “average person,”^37^ and hence, to a better self‐rating.^9^

Most of the results regarding participant characteristics that were associated with UO are in line with findings from previous studies. Individuals with a history of MI were less likely to show UO concerning heart attack risk in our study. Likewise, the very first studies by Weinstein^11^, ^23^ or Helweg‐Larsen and Shepperd^10^ found that personal experience was associated with less prevalent UO.^10^, ^11^, ^23^

Homko et al.^38^ reported that in a sample of individuals with type 2 diabetes, males had a lower comparative risk perception than females when they were asked to compare their CVD risk with others of their age and sex.^36^ In our main analysis, we observed that males were also more likely than females to show UO. However, when the objective comparator was based on a comparison between the calculated individual risk and the mean risk of individuals of the respective age and sex in our sensitivity analysis, this association was no longer statistically significant. Therefore, it is likely that the association in our main analysis resulted from males and females comparing themselves to other individuals of their age and sex, even though the question did not imply this.

Smokers were more likely than nonsmokers to show UO in our study. Strecher et al also reported that smokers were more likely than nonsmokers to show UO.^19^ Furthermore, Ayanian and Cleary reported that many smokers did not perceive themselves at increased MI risk when asked to compare themselves with nonsmokers.^18^ The association between increased blood pressure and UO, which was very robust towards any alterations in our sensitivity analyses, has not been reported in previous studies that examined UO. Therefore, smokers and individuals with higher blood pressure seem to underestimate the increased heart attack risk that results from their respective behavior or characteristic.

The results of our main analysis show that UO and UP were not associated with the measured self‐management behaviors. This was surprising, because theory suggests that UO is a relevant factor in explaining health behavior.^8^, ^10^, ^13^ As Shepperd et al described, we would have expected that individuals who showed UO would show less preventive behaviors, that is, self‐management.^13^ However, our results suggest that UO is not a relevant target when aiming to improve the adherence to self‐management recommendations in individuals with type 2 diabetes.

There are characteristics of our study design that might help explain some of our null results. One explanation could be the domain specificity of UO. Weinstein showed that mean comparative risk judgments varied between different health threats.^23^ Hence, the measure of UO and the outcome of interest need to be directly associated with each other. Five of our self‐management measures, that is, regular self‐monitoring of body weight, blood sugar, and blood pressure; keeping a diabetes diary; and having a diet plan are highly relevant for the prevention of a MI. However, UO with regard to MI might not be representative of an unrealistic risk perception regarding the diabetic foot syndrome. Thus, at least the null association in wound checking could be explained by the health threat specificity of UO. Furthermore, it is possible that a participant is not aware of the association between a behavior and the outcome of interest. Thus, some participants might have been unaware of the link between some of the self‐management behaviors and MI, for example, association between blood sugar testing and MI. Future research should test the participants' awareness of the link between the outcome of interest and the respective behavior. Moreover, there is some critique regarding the Framingham risk equation as the objective comparator. Like other risk equations, for example, United Kingdom Prospective Diabetes Study (UKPDS), the Framingham risk equations have been shown to be only moderately effective in discriminating between individuals at high risk and low risk.^27^ Therefore, some individuals who had been grouped with UO might actually have given an accurate risk estimate and vice versa. However, the main problem reported with regard to the Framingham risk equation has been the overestimation of risk, which does not affect ranking,^27^ and thus does not affect the comparative risk rankings.

We tried to disentangle the association between UO and self‐management behavior from confounding by a positive self‐view. Therefore, we included positive self‐view, that is, self‐rated risk “lower than others,” as an additional covariate in our regression model. The results suggest that UO and positive self‐view have opposing effects on self‐management. Therefore, future studies should consider similar adjustments when examining the association between UO and health behavior.

Our study has several limitations. It is a general concern in surveys that self‐report data suffer from recall bias. However, it is of even greater concern in our study where we based the objective comparator, that is, Framingham risk, on self‐reported data. Nonetheless, a study by Okura et al supports the use of self‐reported information on at least MI and hypertension, as they reported a very high correlation between self‐report and clinical records, that is, 98% and 88%, respectively.^39^ Furthermore, we had no information on the year that the participants had a MI or participated in a disease‐specific education program, so we could not adjust for the time past between these events and data collection. Moreover, person‐positivity bias might have affected the participants' responses to our subjective risk question.^37^ Future studies could consider not making participants compare themselves with an “average person” but with one specifically described comparator that represents an average person. For example, Chock found that comparative optimism with regard to the healthfulness of lifestyle decreased when college students were asked to compare themselves with their best friend.^40^ Another concern is that we assessed MI risk perception while comparing it with the CVD risk. However, due to the similarity of risk factors for MI and CVD and the resulting linearity between the absolute risks for MI and CVD, asking for CVD risk is justifiable.^41^ Finally, our comparative risk question instructed participants to compare their risk with the risk of other patients with type 2 diabetes of their age. Hence, the instruction did not include sex specificity as most of the previous studies did.^9^, ^16^ Accordingly, our main analyses compared the individual comparative risk perception with the ratio between the calculated individual risk and the mean risk of people of the respective age. However, as it is possible that participants compared themselves with peers of the same age and sex, we also estimated the objective comparative risk based on a comparison between the calculated individual risk and the mean risk of individuals of the respective age and sex. Although the overall pattern of associations was qualitatively quite similar, some of the associations of our main analysis were no longer statistically significant. Given this result, we cannot exclude the possibility that some of the participants might have compared themselves with other individuals of their age and sex, even though the comparative risk question did not imply this. Therefore, we would recommend using an age and sex specific question in the future. Another possible issue in our study is selection bias. Of 746 individuals with type 2 diabetes, 113 individuals had missing information that we could not impute. On average, these individuals had a lower education and were more likely to smoke than individuals without missing information. Finally, due to the observational cross‐sectional design of our study, reverse causation and residual confounding cannot be excluded.

One strength of this study lies in the strong theoretic foundation of the methodological approach that takes into account several ideas from previous studies to overcome general issues in the field, for example, the distinction of the positivity of self‐view,^31^ or the issue that individuals with a low comparative risk cannot be grouped with UO. Furthermore, our study includes several sensitivity analyses that allow the study to be compared with most of the previous studies in the field. Other strengths of this study are its large sample size and the detailed information regarding disease‐specific self‐management behavior. Finally, participants of the KORA GEFU 4 study are a random sample from the general population. Therefore, the results are likely to be generalizable for the German diabetes population.

## 5 CONCLUSION

In light of our comprehensive main and sensitivity analyses, we conclude that there are robust associations between smoking status, increased blood pressure, and UO. Thus, participants were likely to underestimate the effects that smoking and high blood pressure have on their heart attack risk. However, we found no significant association between UO and self‐management. Thus, in our sample of patients with type 2 diabetes, targeting UO with regard to heart attack risk would probably not improve the self‐management of the individual.

## CONFLICT OF INTEREST

The authors declare that they have no competing interests.

## AUTHOR CONTRIBUTIONS

Conceptualization: Florian Karl, Rolf Holle, Lars Schwettmann, Michael Laxy

Data Curation: Florian Karl, Rolf Holle, Lars Schwettmann, Annette Peters, Christa Meisinger, Ina‐Maria Rückert‐Eheberg, Michael Laxy

Formal Analysis: Florian Karl, Michael Laxy

Investigation: Florian Karl, Rolf Holle, Lars Schwettmann, Annette Peters, Christa Meisinger, Ina‐Maria Rückert‐Eheberg, Michael Laxy

Methodology: Florian Karl, Rolf Holle, Lars Schwettmann, Michael Laxy

Project Administration: Florian Karl, Michael Laxy

Resources: Annette Peters, Christa Meisinger, Ina‐Maria Rückert‐Eheberg

Supervision: Rolf Holle, Lars Schwettmann, Michael Laxy

Visualization: Florian Karl

Writing – Original Draft Preparation: Florian Karl, Rolf Holle, Lars Schwettmann, Michael Laxy

Writing – Review & Editing: Florian Karl, Rolf Holle, Lars Schwettmann, Annette Peters, Christa Meisinger, Ina‐Maria Rückert‐Eheberg, Michael Laxy

All authors have read and approved the final version of the manuscript. Florian M. Karl had full access to all of the data in this study and takes complete responsibility for the integrity of the data and the accuracy of the data analysis.

## TRANSPARENCY STATEMENT

Florian M. Karl affirms that this manuscript is an honest, accurate, and transparent account of the study being reported; that no important aspects of the study have been omitted; and that any discrepancies from the study as planned (and, if relevant, registered) have been explained.

## Data Availability

The data that support the findings of this study are available from KORA (https://www.helmholtz-muenchen.de/en/kora/for-scientists/cooperation-with-kora/index.html) but restrictions apply to the availability of these data, which were used under license for the current study, and so are not publicly available. However, data can be requested through an individual project agreement with KORA via the online portal KORA.passt (https://epi.helmholtz-muenchen.de/).
